# Unveiling microbiome profiles in human inner body fluids and tumor tissues with pancreatic or biliary tract cancer

**DOI:** 10.1038/s41598-022-12658-8

**Published:** 2022-05-24

**Authors:** Shujiro Okuda, Yuki Hirose, Hayato Takihara, Akiko Okuda, Yiwei Ling, Yosuke Tajima, Yoshifumi Shimada, Hiroshi Ichikawa, Kazuyasu Takizawa, Jun Sakata, Toshifumi Wakai

**Affiliations:** 1 Division of Bioinformatics, Niigata University Graduate School of Medical and Dental Sciences, 2-5274 Gakkocho-dori, Chuo-ku, Niigata, 951-8514 Japan; 2 Medical AI Center, Niigata University School of Medicine, 2-5274 Gakkocho-dori, Chuo-ku, Niigata, 951-8514 Japan; 3 Division of Digestive and General Surgery, Niigata University Graduate School of Medical and Dental Sciences, 1-757 Asahimachi-dori, Chuo-ku, Niigata, 951-8510 Japan; 4 Department of Medical Technology, Graduate School of Health Sciences, Niigata University, 2-746 Asahimachi-dori, Chuo-ku, Niigata, Niigata 951-8518 Japan

**Keywords:** Oncology, Microbiome, Computational biology and bioinformatics

## Abstract

With the discovery of bacterial symbiosis in the tissues of various cancers, the study of the tumor microbiome is attracting a great deal of attention. Anatomically, since the gastrointestinal tract, liver, and pancreas form a continuous ductal structure, the microbiomes in the digestive juices of these organs may influence each other. Here, we report a series of microbiome data in tumor-associated tissues such as tumor, non-tumor, and lymph nodes, and body fluids such as saliva, gastric juice, pancreatic juice, bile, and feces of patients with pancreatic or biliary tract cancers. The results show that the microbiome of tumor-associated tissues has a very similar bacterial composition, but that in body fluids has different bacterial composition which varies by location, where some bacteria localize to specific body fluids. Surprisingly, *Akkermansia* was only detected in the bile of patients with biliary tract cancer and its presence was significantly associated with the performance of external biliary drainage (*P* = 0.041). Furthermore, we found that tumor-associated tissues and body fluids in deep inner body are mostly inhabited by unidentified and uncharacterized bacteria, suggesting that such bacteria may be potential targets for precision therapy in the future.

## Introduction

Metagenomic analysis that comprehensively sequences whole DNAs from bacteria that are symbiotic with the human body, has been widely implemented^1,2^. Knowledge about the phylogenetic and genomic composition of bacteria that are symbiotic in a lot of human body sites, such as the intestines and skin, has been accumulated. In addition, it has been demonstrated from various viewpoints that symbiotic bacteria affect human diseases and health^3–7^. However, most of these studies target bacteria that live on the surface of the human body, in the sense of its intestinal epidermis even if it is in the gut^1,8,9^. On the other hand, it has recently been reported that specific bacteria found inside tumor tissues would be resistant to anti-cancer drugs and also affect the prognosis^3,10,11^. Bacteria inside the human body, such as in tissues and cells, have attracted a lot of attention; especially the bacteria found in tumor tissues are being regarded important and studied under a new field, called tumor microbiome^10,12,13^.

Anatomically, the gastrointestinal tract, liver, and pancreas form a continuous luminal structure. Therefore, the microbiomes in the digestive juices of these organs may interact with each other. Since it is relatively easy to collect sample, microbiome analysis from saliva, gastric juice, and feces has often been performed under different conditions. The intestinal microbiome has been analyzed and implied the associations between bacteria and colorectal cancer and many other variety of diseases^14–16^. In addition, gastric juice microbiome analysis has been conducted and been found of clinical significance, suggesting its association with gastric cancer^17–20^. However, it has been difficult to investigate the tissues or body fluids from other inner part in the human body, such as bile and pancreatic juice, due to the high invasiveness of the procedure required for sample collection.

Biliary tract and pancreatic cancer are known as ‘carcinomas with a poor prognosis in the digestive tract’^21–23^. Growing evidence suggests that microbiomes can influence the efficacy of cancer therapies^3,11^. In the future, it is expected that such accumulation of knowledge will contribute to the improvement of treatment outcomes for intractable cancers, such as biliary tract cancer and pancreatic cancer.

Here, we report a series of microbiome datasets in tumor-related tissues including tumor, non-tumor, and lymph node with or without metastasis, in addition to body fluids of saliva, gastric juice, pancreatic juice, bile, and feces from pancreatic or biliary tract cancer patients. We found that microbiomes in tumor-related tissues had very similar composition of bacteria, but those in body fluids have different bacterial compositions depending on their location; some bacteria are localized in a specific body fluid. In addition, we found that tumor-related tissues and the other body fluids apart from saliva, gastric juice, and bile, were occupied by uncharacterized bacteria that can only be identified by the family taxonomic level. These facts suggest that the bacteria living in body fluids or inner body tissues would possibly have many unknown organisms that have not yet been isolated and identified and such bacteria would be potential targets for future precision therapy.

## Methods

### Sampling

Fifteen patients who underwent curative resection for biliary tract cancer (N = 11) or pancreatic cancer (N = 4), from August 2018 through October 2018 at the Niigata University Medical and Dental Hospital, were enrolled in this study. These patients included 10 men and 5 women, with a median age of 66 (42–87) years. Seven of them had lymph node metastasis, and 8 had microscopic lymphovascular invasion. Clinicopathologic characteristics of the 15 patients are shown in Table 1 and Supplementary Table 1.

**Table 1:** Clinicopathologic characteristics of 15 patients with biliary tract or pancreatic cancer.

Variable		No. of patients
Age (years)*		66 (42–87)
Gender	Male	10
	Female	5
Preoperative biliary drainage	Absent	4
	Present	11
Type of cancer	Biliary tract cancer	11
	Pancreatic cancer	4
Lymph node metastasis	Absent	8
	Present	7
Lymphovascular invasion	Absent	7
	Present	8

		Concentration
Maximum T-Bil level (mg/dL)*		2.6 (0.4–17.8)
Serum CEA level (ng/mL)*		4.5 (1.6–13.5)
Serum CA19-9 level (U/mL)*		58 (1–11,277)

T-Bil, total bilirubin; CEA, carcinoembryonic antigen; CA19-9, carbohydrate antigen.

*Data are expressed as median (range).

All patients received sodium picosulfate hydrate the day before the elective surgery. Broad-spectrum antibiotics was used during the surgery and for 0–2 days postoperatively. During the operation, samples of saliva, gastric fluid, and feces were retrieved from the patients just after anesthesia, by suction or digital examination of the anus. Bile samples were retrieved from endoscopic nasal biliary drainage tube or percutaneous transhepatic biliary drainage tube, preoperatively or from surgically resected specimen. Pancreatic fluid was sampled the day after surgery from pancreatic drainage tube, which was intraoperatively inserted into the pancreatic duct of the remnant pancreas. Tumor tissues, non-tumor tissues, and metastatic lymph nodes were sampled from the surgically resected specimen immediately after surgery. All samples were stored at − 80 ℃ immediately after retrieval. Categorical variables (clinicopathologic characteristics of patients with biliary tract cancer and *Akkermansia* status of bile samples) were compared using Fisher’s exact test. This retrospective analysis was performed in accordance with the Helsinki Declaration, and the Ethics Committee on Genetic Analysis of Niigata University approved the study protocol (G2020-0038). Informed consent for this study was obtained from all patients.

### 16S rRNA gene sequence

16S rRNA sequences were sequenced in each body fluid and tissue by the Mykinso® technology manufactured by Cykinso Inc., which included DNA extraction and the 16S rRNA paired-end sequencing that followed. The obtained FASTQ file was combined with a paired end DNA read as a single read sequence using fastq-join with default settings. Next, according to the method of QIIME (Quantitative Insights Into Microbial Ecology) version 1, quality check and chimera sequence filter were performed. Relative abundance was calculated after detecting an operational taxonomic unit (OTU) at 97%. The OTU level for the filtered sequence data using QIIME’s “pick_open_reference_otus” command. The number of reads in each step is listed in Supplementary Table 2.

### Contaminant filtering and re-calculation of relative abundance

Recent advances have shown that bacteria exist in a variety of tumor tissues, and these studies have also suggested that contamination may occur during the process of tissue sampling and subsequent processing. We performed the removal of contaminants, described in a previous report^10^, from the species obtained in this study. The species name that completely matched the contaminants were filtered out and then we re-calculated the relative abundance of the remaining species after normalized to 1.0. The relative abundance at the upstream taxonomy levels was re-estimated based on re-calculated relative abundance at the species level.

### Hierarchical clustering

Genus with a relative abundance of 1% or more in the tumor tissue were filtered. In addition, genus undetected with more than 95% of the samples were also filtered. Hierarchical clustering was performed on the relative abundance of remaining genus with the Euclidean distance and the Ward method in R (https://www.r-project.org). Similar clustering was performed for OTUs with more than 1% relative abundance in the tumor tissue.

### Similarity between microbiome

The microbiome similarity between each sample was calculated using the Dice index, according to the method reported by Moeller et al.^24^. OTUs with more than 0.01% relative abundance, found in more than one sample, were extracted and the similarity between samples (A and B) were calculated by the following formula:

$${\text{Dice index }} = { 2}\left( {\left| {\text{A}} \right| \cap |{\text{B}}|} \right)/\left( {\left| {\text{A}} \right| + |{\text{B}}|} \right)$$

## Results

### Uncharacterized bacteria found in the inner body sites

**Figure 1:**
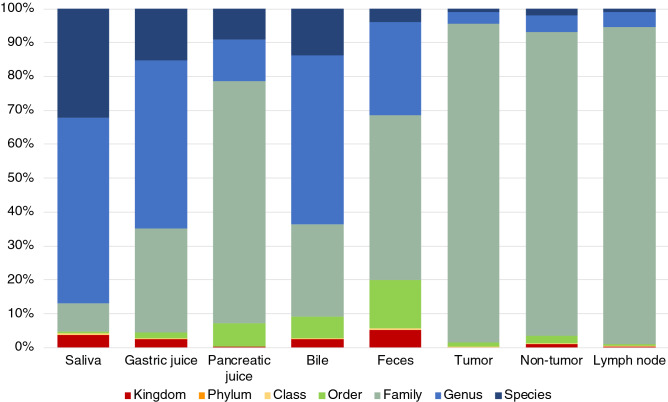
Coverage of taxonomy at each body fluid and tissue. The average relative abundance of taxonomy for patients at each site was used as coverage against the total abundance.

At each inner body site, we investigated how much abundance of bacteria in each taxonomic level was identified (Fig. 1 and Supplementary Table 1). As a result, in saliva, the 16S rRNA gene sequences that can be identified up to genus and species level accounted for about 90% of the total, suggesting that there exist a lot of well-known bacteria in the site. On the other hand, in tumor, non-tumor and lymph nodes, more than 90% of the sequences were only identified at the family level, implying that most of occupied bacteria were unknown and uncharacterized. Although, about 60% bacteria in gastric juice and bile were identified at the genus and species levels, suggesting these samples were close to saliva sample, it was suggested that uncharacterized bacteria probably prevail in pancreatic juices and feces.

### Microbiomes from inner body fluids and tumor-related tissues

**Figure 2:**
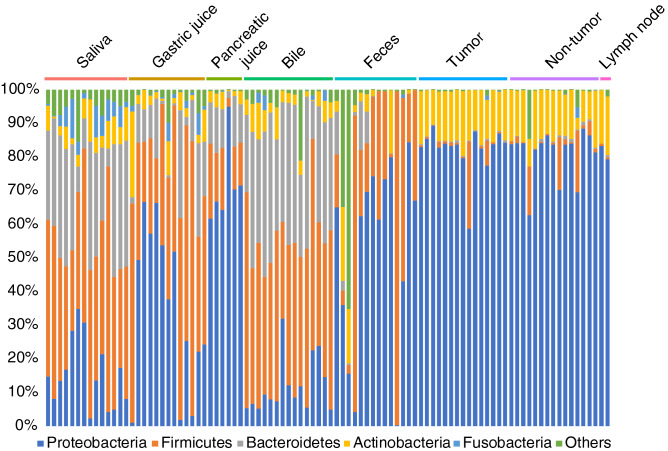
Profiles of symbiotic microbiome in the body fluids and tissues at phylum level. Site-specific distribution of relative abundance of each phylum are shown.

**Figure 3:**
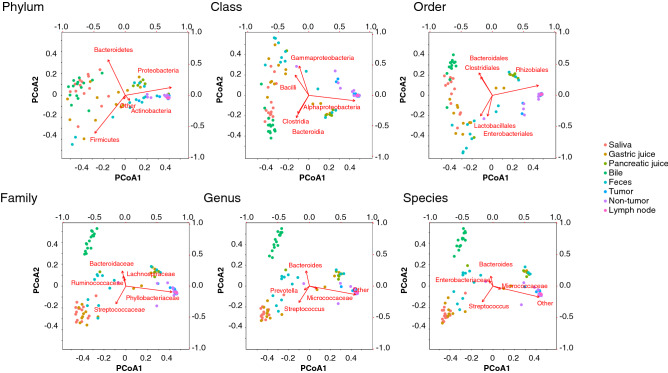
PCoA in each taxonomy level. Calculation was based on the relative abundance at each taxonomic level.

Samples from some inner body fluids including saliva, gastric juice, pancreatic juice, bile, and stool, and tumor-related tissues including tumor, non-tumor, and lymph node, were collected from biliary tract cancer or pancreatic cancer patients. In some cases, it was not possible to obtain samples at specific sites and samples from all sites were not obtained for all patients. DNA was extracted from the obtained samples and 16S rRNA genes were sequenced. The resultant 16S rRNA gene sequences were processed by the QIIME method, and the relative abundance in each taxonomy was calculated. Figure 2 shows the distribution of relative abundance at the phylum level in each sample. The phylum level taxonomy was differently distributed in each body site. Saliva and bile were likely to have less Proteobacteria and more Firmicutes, and tumor-related tissues had similar distribution patterns. On the other hand, phylum in gastric juice and pancreatic juice showed the reverse tendency. There were very little Firmicutes and dominant Proteobacteria in tumor, non-tumor, and lymph node samples. Furthermore, in these sites, Actinobacteria was likely to be more abundance than the other body fluids samples. Similarity between samples in this phylum level was found to be similar with other taxonomic levels. In addition, PCoA analyses was performed in each taxonomy level (Fig. 3). From higher to lower taxonomy level, each site was clearly distinguished.

### Localization of microbiome to each inner body sites

**Figure 4:**
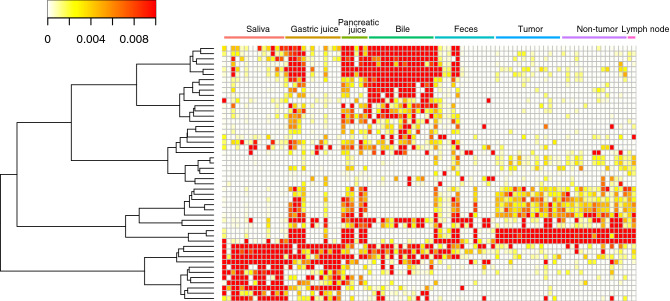
Clustering using Genus-level relative abundance. Clearly clustered areas are shown from the results of clustering using only genera with relative abundance of more than 1% (Supplementary Fig. 2). The relative abundance values were clustered using Euclidean distance and Ward’s method.

Clustering was performed based on the values of relative abundance in genus level, that is more granular taxonomy level. As a result, it was shown that genus with a certain level of the relative abundance has differently localized in the inner body sites (Fig. 4 and Supplementary Fig. 1). It was found that bacteria in saliva were often found in the gastric juice and bile of the patients. Bacteria found in gastric juice samples were also found to colocalize with pancreatic juice and bile. The same bacteria are colocalized in tumor, non-tumor, and lymph node samples and these bacteria are also likely to be present in gastric juice, pancreatic juice, bile and be part of feces. In addition, we found that some bacteria were specifically detected in each body site (Supplementary Table 3). Of these bacteria found only at each inner body site, *Akkermansia* had the highest average abundance and was found only in bile samples. In the samples used in this study, *Akkermansia* was detected in 7 out of 11 patients with biliary tract cancer but was not detected in bile samples from 4 patients with pancreatic cancer (*P* = 0.077). Statistical tests were performed to determine whether this bile-specific *Akkermansia* was associated with the clinical background of all 15 patients. It was detected in 6 out of 8 (75%) patients with external biliary drainage, whereas only in 1 out of 7 (14.3%) patients without external biliary drainage. Thus, presence of bile-specific *Akkermansia* was significantly associated with the performance of external biliary drainage (*P* = 0.041).

### Origin of the bacteria found in tumor tissues

In order to investigate which tissue/fluid from which the bacteria found in tumor tissue originate, the similarity of the microbiome between each sample was compared. The similarities between saliva and gastric juice, and tumor and non-tumor tissues showed higher values than other combinations (Supplementary Fig. 2). Similarity between bile and the other sites was rather low. The distribution of similarity between the inner body sites in the same patient was different from each other. On investigating the similarity with the other sites from tumor tissue it was found that, non-tumor tissue showed the highest but moderate similarity values, and the next highest similarity was in fecal sample (Supplementary Fig. 3). In addition, there was no clear similarity between each site in biliary tract cancer and pancreatic cancer patients.

### Comparison of the representative genus from tumor tissue

**Figure 5:**
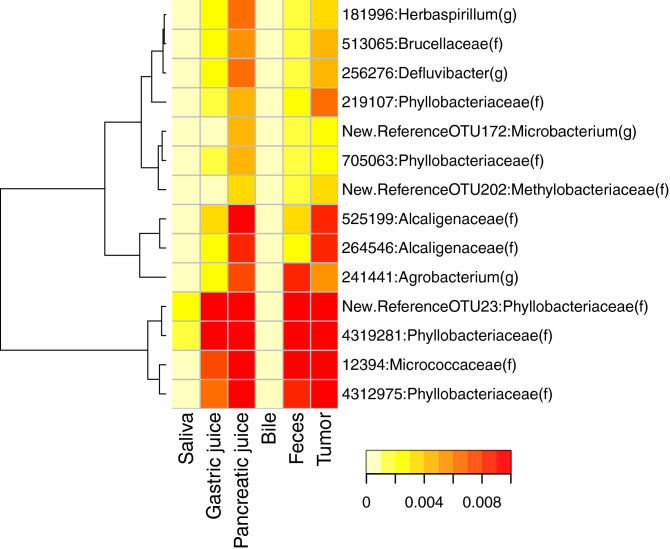
Bacterial origin in tumor tissue. OTUs with a relative abundance > 1% in the tumor tissue were extracted from the other body fluids and tissues. The relative abundance values of the OTUs were clustered using Euclidean distance and Ward’s method.

Clustering was performed to examine which tissues/fluids co-localize with OTUs, with 1% or more relative abundance in tumor tissue (Fig. 5). As a result, it was found that bacteria often present in the tumor tissue were not likely to be found in saliva and bile, but are co-localized in pancreatic juice, gastric juice and feces. In particular, the representative bacterial genus found in pancreatic juice strongly co-localized in tumor tissue.

## Discussion

The concentration of bacteria tends to increase from the oral cavity to the stomach and intestinal tract and is said to range from 10^2^ to 10^12^^25^. In the case of body fluids, such as pancreatic juice and bile, which we have studied here, the number of bacteria is expected to be very low due to the presence of very high concentrations of digestive enzymes and the extreme environment of stably high pH. However, the results of this study indicated that bacteria are also present in these body fluids, although the results are limited to specific cancer patients. This suggests the possibility of symbiosis with bacteria in the deep inner body environment, where the presence of bacteria has not previously been noticed. The presence of bacteria inside cancer tissues has been shown to affect the efficacy of anticancer drugs and the prognosis of patients, depending on the activity of these bacteria^26^, and it cannot be denied that these symbiotic bacteria may have some effect on the host human metabolic system in body fluids other than cancer tissues. In addition, our results showed that the localizations of bacteria in each body fluids and tissues were different (see Fig. 4). This could be the result of adaptation of the bacterial community to respective environments. Although the pancreas and bile are alkaline with a high pH and gastric juice is an acidic environment with a low pH, bacteria that exist in these special environments are presumed to be able to survive because they have metabolic functions that are suitable for these environments. However, most of the bacteria detected in the environment, such as tumor tissue, pancreatic juice, and bile, were uncharacterized bacteria that could not even be identified at the genus level (see Fig. 1), and it is extremely difficult to infer their actual biological functions. Future large-scale gene function analyses, such as comprehensive metagenome analysis, will be necessary to elucidate their functions.

*Akkermansia* had the highest average abundance and was found only in bile samples. In the samples used in this study, *Akkermansia* was detected in 7 out of 11 patients with biliary tract cancer but was not detected in bile samples from 4 patients with pancreatic cancer (*P* = 0.077). Statistical tests were performed to determine whether this bile-specific *Akkermansia* was associated with the clinical background of all 15 patients; bile-specific *Akkermansia* was detected in 6 out of 8 (75%) patients with external biliary drainage, whereas it was detected in only 1 out of 7 (14.3%) patients without external biliary drainage. Therefore, presence of bile-specific *Akkermansia* was significantly associated with the performance of external biliary drainage (*P* = 0.041).

Majority of bacteria were uncharacterized in specific body fluids and tissues, but this may be due to a bias in the 16S rRNA database used. We also tried including Ribosome Database Project (RDP), but the results were almost the same. In addition, the microbiome inside tumor tissues is a topic that has been attracting attention in the past few years. Therefore, the deep inner body environments have not been the subject of microbiome studies, and the results suggest that there are many unknown bacteria in such an environment that have not yet been elucidated even at genus level. However, the clinical significance of the fact that bacteria are present in human tissues and body fluids, where the presence of bacteria has not originally been paid attention to, is significant. Based on our findings, the microbiome inside the body deserves attention as a target for the treatment of cancer.

The bacteria that coexist in the tumor tissue are relatively common to those localized in pancreatic and gastric juice (see Fig. 5), suggesting that they may originate from these environments, but there are also many individual differences (Supplementary Fig. 2). When comparing tissues and body fluids within each individual, saliva and gastric juice showed a high degree of similarity, but other digestive tissues showed different similarities, suggesting dynamic changes in the composition of the bacterial flora that adapted to each environment (Supplementary Fig. 3). With regard to the OTUs that were relatively abundant in the tumor tissue, these were common with OTUs in gastric and pancreatic juices (compared to that in other body fluids and tissues). However, bile tended to have only a few OTUs in common, suggesting that the body’s internal environment may be very special in terms of composition of the microbiome (see Fig. 5). Bacteria symbiotic with tumor tissue may be derived from gastric juice and pancreatic juice, which have many common OTUs.

The main limitations of the current study include the retrospective nature of the analysis (e.g., administration of antibiotics, approach of biliary drainage, and pancreatic drainage), the small number of patients analyzed, and variability in the timing of sample collection. Pre-operative broad-spectrum antibiotic administration, the types of drainage of body fluids, and the sampling time might affect microbiome composition and diversity. Most of those limitations come from the difficulty of sampling the fluids existing in the body deep inside. In addition, this study required sampling multiple tissues and body fluids from each individual patient, which limited the amount and frequency of sampling. The number of patients who could be compared was also limited as there are not many patients eligible for sampling from multiple environments. Therefore, the number of patients who could be used in this study was limited to 15 patients for pancreatic and biliary tract cancers, and since the results obtained in this study are based on such a limited number of patients, they may not represent all cancer patients. Nevertheless, we believe that our findings are clinically meaningful, and it is hoped that similar comparative analyses can be performed in larger cohorts in the future.

Finally, the present analysis did not lead to the discovery of bacterial markers that could be applied to the treatment or prevention of cancer, but since *Phylobacteriaceae* were dominant in the tumor tissues of several patients, the search for the clinical significance of this bacterial genus in pancreatic and biliary tract cancer may be of significance for the future. Cancer treatment involves the use of targeted precision medicine, such as molecular targeted drugs; therefore, the presence or absence of bacteria in tumor tissues and the effect of differences in intestinal microbiome on the therapeutic effect have been studied^3,10,11^. In the future, it will be necessary to develop treatment methods that also focus on bacteria inside tumor tissues and/or body fluids.

## Conclusions

We found that bacteria were detected in all samples, i.e., not only from tumor-associated tissues but also from body fluids such as gastric juice, pancreatic juice, and bile. In tumor-associated tissues and pancreatic juice, many 16S rRNA gene sequences were detected, which have not been unidentified so far, suggesting that bacteria thriving deep inside the human body may constitute a hitherto unknown world that has not yet been studied. Since *Akkermansia* was only detected in bile, the fact that this particular bacterial species is enriched in certain tissues or inner body fluids, as well as the fact that bacteria inside the tumor tissues correlate with gastric and pancreatic juices, could be of use for exploring potential new therapeutic targets.

## Supplementary Information

Supplementary Information 1.
Supplementary Figure 1. Clustering using Genus-level relative abundance more than 1%. The relative abundance was clustered with Euclidean distance and Ward’s method.

Supplementary Information 2.
Supplementary Figure 2. Similarity of OTU profiles between each site in individuals. The similarity between each site was calculated using the Dice index (see Methods). The OTUs with the relative abundance with more than 0.01% were used for the calculation.

Supplementary Information 3.
Supplementary Figure 3. Similarity of microbiome profiles in tumor tissue to other sites in each patient. The relative abundance of OTUs were clustered with Euclidean distance and Ward’s method.

Supplementary Information 4.
Supplementary Table 1. Sample information, and relative abundance in each taxonomy used in this study.

Supplementary Information 5.
Supplementary Table 2. Read mapping information.

Supplementary Information 6.
Supplementary Table 3. Site specific detected taxon.

## Data Availability

The datasets generated and/or analysed during the current study are available in the DDBJ DRA repository^27^, PRJDB13257.
