# Supplementary figures and images for: Unveiling microbiome profiles in human inner body fluids and tumor tissues with pancreatic or biliary tract cancer
Source: Sci Rep. 2022 May 24;12:8766. doi: 10.1038/s41598-022-12658-8 (PMC9130259; doi:10.1038/s41598-022-12658-8)

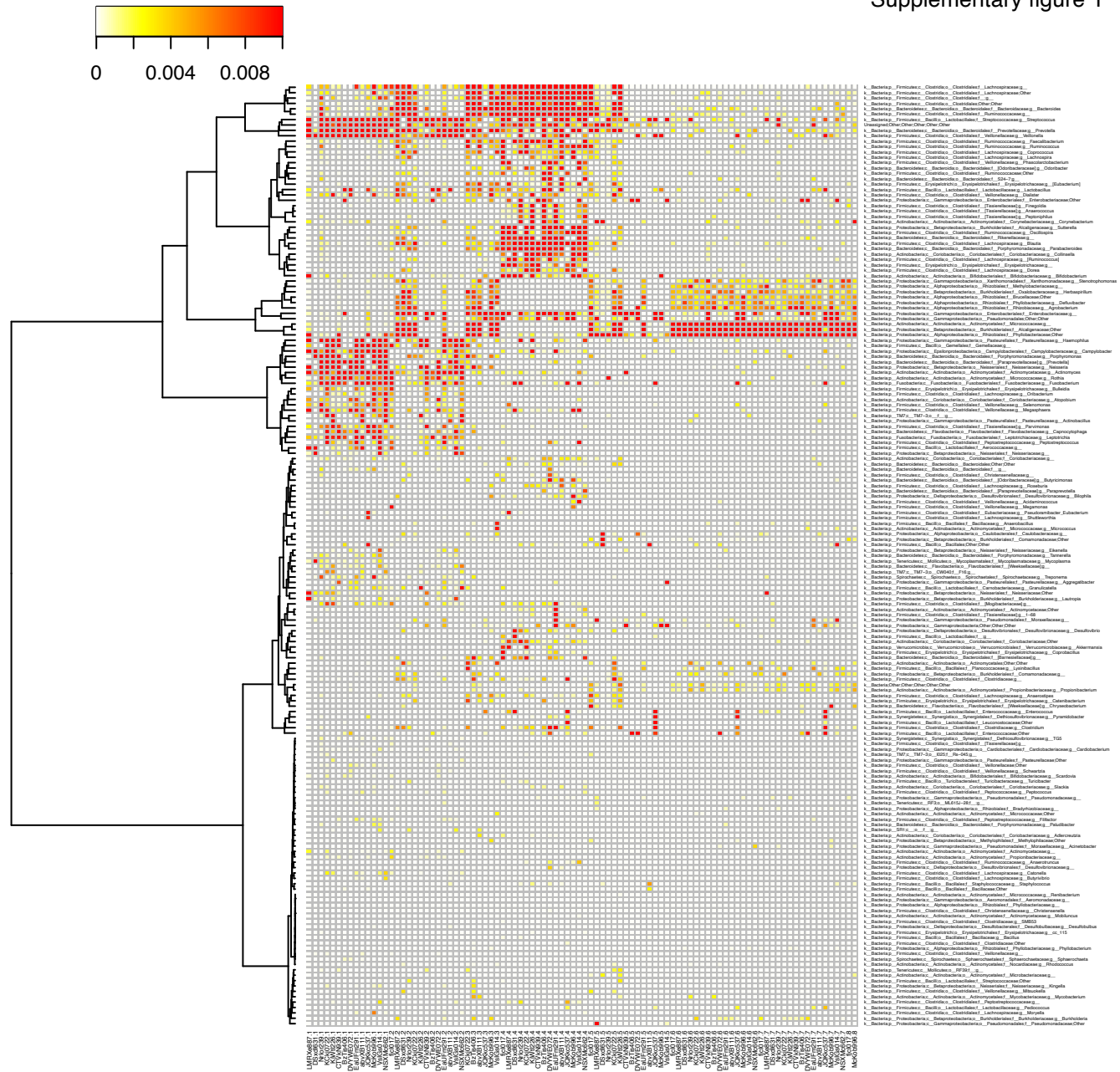

Supplement: Supplementary file 1 — Supplementary Information 1. Supplementary Figure 1. Clustering using Genus-level relative abundance more than 1%. The relative abundance was clustered with Euclidean distance and Ward’s method. [file 41598_2022_12658_MOESM1_ESM.pdf]

Biliary tract cancer

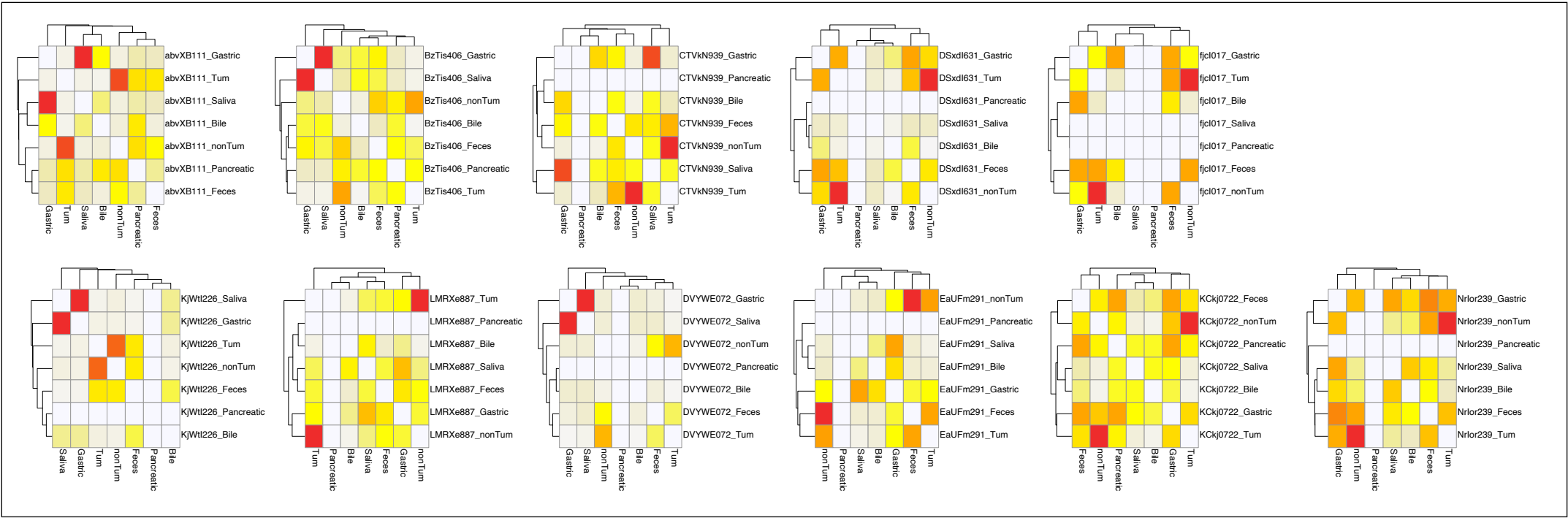

Pancreatic cancer

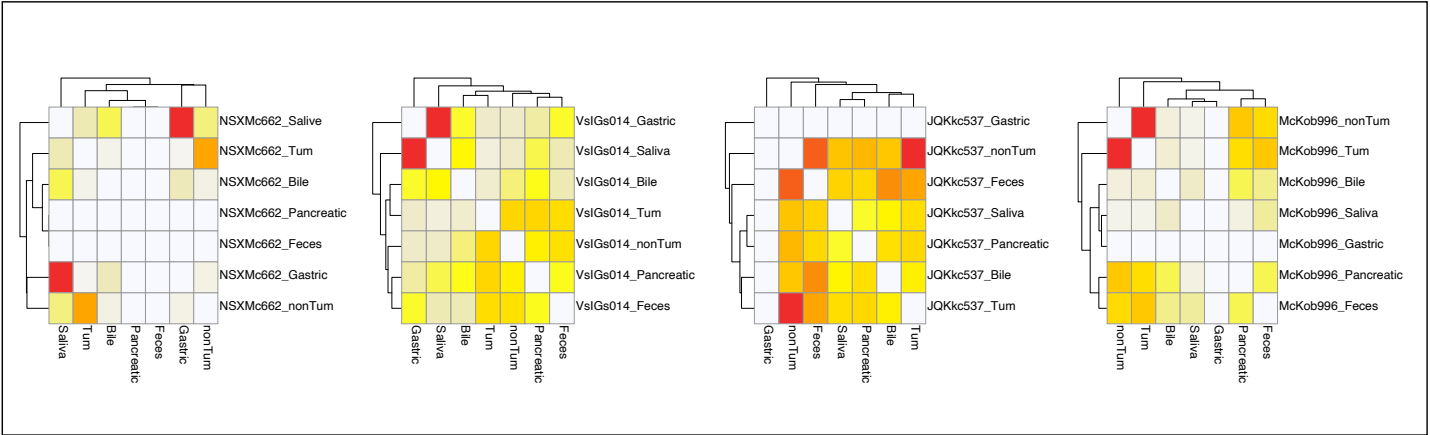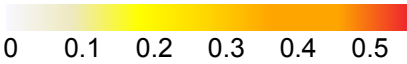

Supplement: Supplementary file 2 — Supplementary Information 2. Supplementary Figure 2. Similarity of OTU profiles between each site in individuals. The similarity between each site was calculated using the Dice index (see Methods). The OTUs with the relative abundance with more than 0.01% were used for the calculation. [file 41598_2022_12658_MOESM2_ESM.pdf]

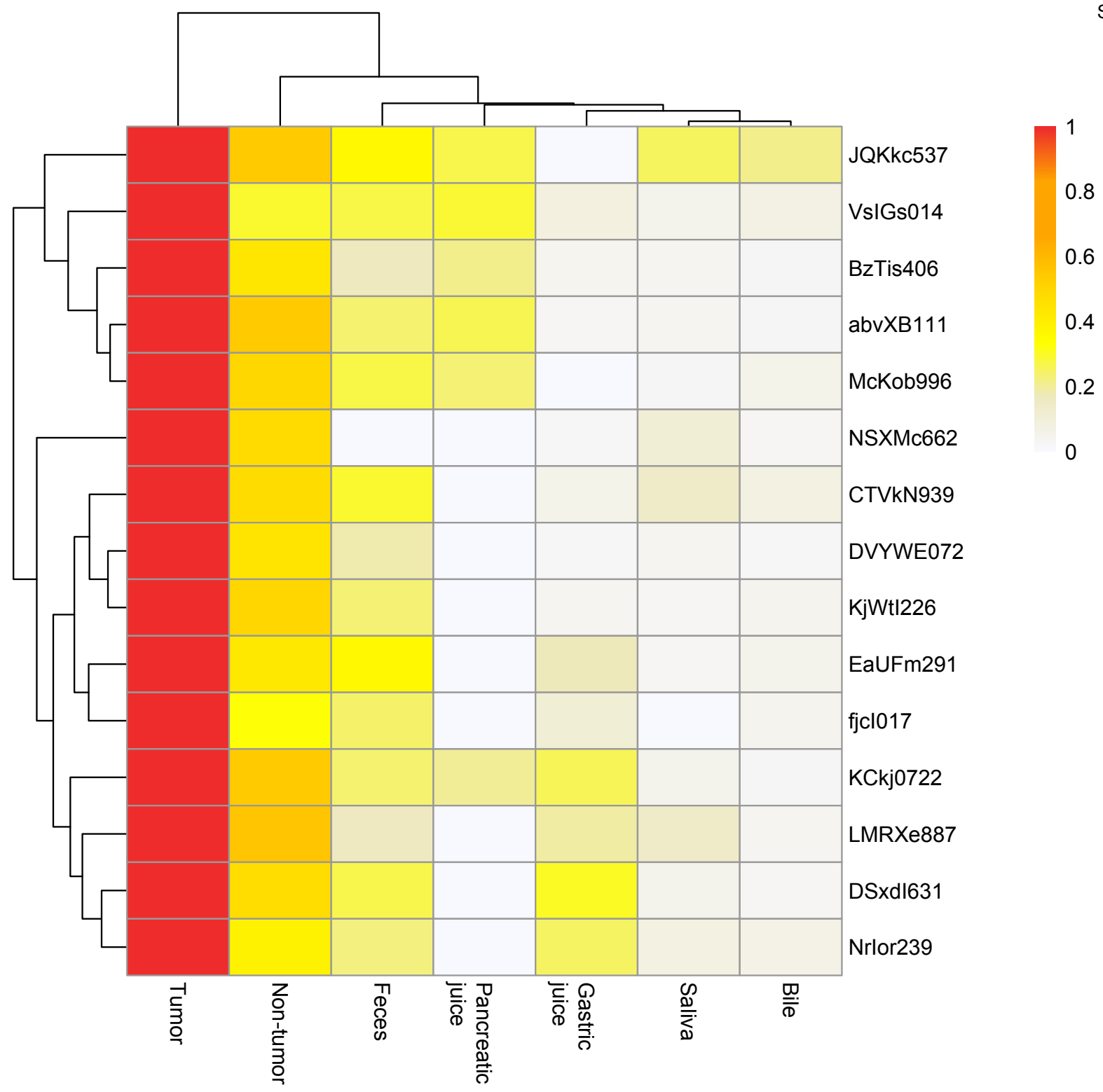

Supplement: Supplementary file 3 — Supplementary Information 3. Supplementary Figure 3. Similarity of microbiome profiles in tumor tissue to other sites in each patient. The relative abundance of OTUs were clustered with Euclidean distance and Ward’s method. [file 41598_2022_12658_MOESM3_ESM.pdf]
